# Hotspot mutations in HER2 interfaces destabilize structure, causing breast cancer treatment failure

**DOI:** 10.21203/rs.3.rs-5931887/v1

**Published:** 2025-03-20

**Authors:** Abhijit De, Pranay Dey, Aniketh Bishnu, Janvi Patel, Shivali Mishra, Nikhil Gadewal, Pritha Ray, Sudeep Gupta

**Affiliations:** Advanced Centre for Treatment Research & Education in Cancer; Advanced Centre for Treatment, Research and Education in Cancer

**Keywords:** breast cancer, hotspot mutations, HER2-positive, dimerization domain mutations, trastuzumab, neratinib, treatment resistance

## Abstract

Many HER2-positive breast cancer (BC) patients relapse within a year of trastuzumab or neratinib treatment. We identified specific pathogenic mutations in the dimerization domains II and IV of the HER2 receptor that contribute to treatment resistance. Mutations G309A, S310Y, and P523S induce significant structural alterations, disrupting crucial HER2:HER2 binding pockets. HER3-preferring mutants exhibited increased HER2:HER3 interactions, as confirmed by proximity ligation assay in HER2-low and HER2-high cell lines. G309A, S310Y, and P523S mutations induced a receptor switch, altering downstream signaling from ERK to AKT activation, leading to high insensitivity to trastuzumab or neratinib in cell survival and migration assays, which was further confirmed by bioluminescence imaging of orthotopic tumors expressing the P523S mutation. This study identifies new hotspot mutations in HER2 domains II and IV causing trastuzumab resistance. Notably, cells with either wild-type or the examined dimerization domain mutations retained sensitivity to the FDA-approved HER2 kinase inhibitor, tucatinib.

## Introduction

Breast cancer (BC) is the most prevalent malignancy in women worldwide, with approximately 1.67 million new cases diagnosed in 2022, constituting 30% of all cancer diagnoses.^1^ While a minority of patients with BC initially present with metastatic disease, up to one-third of those with early-stage disease subsequently develop resistance and metastasis. The human epidermal growth factor receptor 2 (HER-2/neu) overexpression is observed in approximately 25–30% of all BC cases.^1–4^ HER2 receptor, a tyrosine kinase and oncogene, promotes oncogenesis through gene amplification and/or mutations. Ligand binding induces HER2:HER2 homodimer formation, primarily involving domain II (critical for dimerization) and domain IV (stabilizing the receptor complex). Subsequent receptor phosphorylation triggers signal transduction to downstream candidates, promoting cancer cell proliferation.^5^ Various HER2-targeted cancer drugs, including trastuzumab (rst-line therapy), neratinib, pertuzumab, and other second/third-line therapy, are available for treating this subtype of patients.^3,4,6,7^

Trastuzumab, a humanized recombinant monoclonal antibody-based drug, which demonstrated a 33% reduction in death risk for patients with HER2-positive BC, is a pivotal achievement in the personalized cancer medicine sector.^8^ However, effective response to trastuzumab-based therapy in advanced disease is limited to 5–9.1 months, indicating widespread acquired resistance.^9^ Reports predominantly suggest HER2 downstream signaling over-activation due to activating mutations, altered signaling cascades, and immune evasion by the cancer cells.^10,11^ HER3 is crucial for signaling activation in HER2-overexpressing BC.^12,13^ Additionally, studies evaluating siRNA-mediated disruption of HER2:HER3 dimerization, which impacts downstream PI3K-AKT signaling, highlighted the key role of HER3 pseudo-kinase in mediating resistance to gefitinib or lapatinib.^14^ Furthermore, IGF-1R and HER2 interaction has been implicated in trastuzumab resistance in HER2-positive BC cells.^15^

The evolving dynamics of HER2 interactions with other receptor tyrosine kinases (RTK) responsible for the onset of chemo-resistance represent a relatively recent and compelling topic in BC research. Lapatinib resistance often involves a switch from HER2:HER2 homodimer to EGFR:HER3 heterodimer, altering the signaling cascade.^10^ Subtype switching from HER2-positive to estrogen and/or progesterone receptors-positive has been linked to therapy resistance.^16,17^ Reports have suggested the oncogenic nature of some HER2 mutations.^17,18^ However, therapeutic attention has been primarily focused on tyrosine kinase domain mutations, such as L755S and V777L. A recent study that evaluated the consequences of these mutations when co-occurring with HER3-E928G^19^ revealed pronounced activation of the PI3K-AKT signaling axis driven by HER3, thereby causing resistance to HER2-targeted therapeutics. The BC cells harboring these mutations were highly susceptible to pharmaceutical PI3K inhibition. Moreover, we have reported alterations in the conventional HER2:HER3 interactions due to HER3 dimerization domain mutations (DDMs).^20^ For instance, HER3-D297Y was found to preferentially interact with EGFR, which can be targeted by different lines of EGFR therapeutics. Similarly, in lung cancer, HER4 DDMs were reported to induce receptor switching from HER4:HER4 homodimer to HER4:HER2 heterodimer.^21^ While HER2:HER2 homodimer activates MAPK signaling in the absence of ligands, HER3 preferably forms heterodimer with HER2 in the presence of Neuregulin (NRG-1) and NRG-2, but possesses impaired kinase activity.^2,22^ However, phosphorylation by HER2 triggers oncogenic signaling via PI3K/AKT pathway.^12,23–25^

Pathogenic mutations affecting the domains crucial for HER2 interaction with other RTK remain unexplored in the context of drug resistance. Additionally, the activation or incomplete inhibition of a receptor in the receptor dimer complex has been shown to enhance drug resistance.^2,5,12^ Therefore, mutations in the HER2 dimerization domains could alter receptors’ binding partner, leading to incomplete inhibition of downstream signaling response. A recent study highlighted the tendency to form HER2:HER3 heterodimer instead of HER2:HER2 homodimer in the presence of S310F, the only DDM studied so far, with HER3 stabilizing the S310F-HER2 interaction via hydrogen-bonding.^3^

In light of these findings, we aimed to elaborate on the structural, molecular, and therapeutic implications of HER2 DDMs present in HER2-positive BC. Thus, we searched global BC databases, identified hotspot DDMs of HER2, and performed in-depth in silico analysis to discern structural alterations induced by these mutations. Since significant structural changes were observed, we performed extensive investigation on molecular, phenotypic, and therapeutic consequences using relevant assessments in BC cell lines and preclinical model.

## Results

### Hotspot mutations in HER2 dimerization domains induce conformational changes in the receptor’s structure

**Figure 1:**
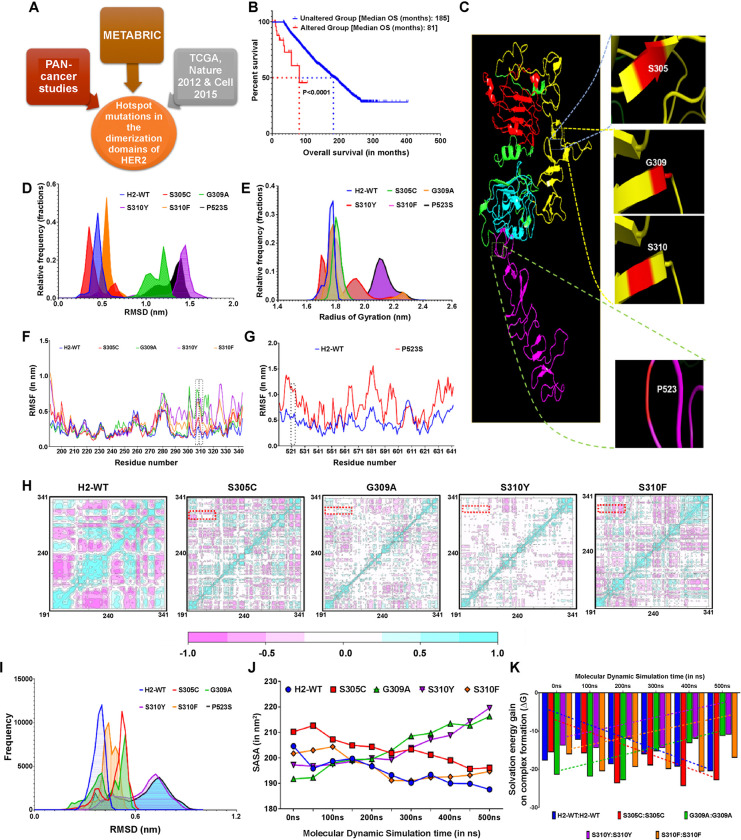
Structural and mechanistic insights into hotspot HER2 dimerization domain mutations (DDMs). (**A**) Schematic representing the analysis pipeline of The Cancer Genome Atlas BC cohorts to identify the hotspot HER2 DDMs. (**B**) Kaplan–Meier curves comparing the overall survival between patients with wild-type HER2 (H2-WT) breast cancer (BC) (unaltered group) and those with HER2 DDMs (altered group) demonstrated a hazard ratio of 0.04803 (95% confidence interval (CI): 0.01193–0.1934). (**C**) 3D protein astructure model of HER2 in open form showing location of the five identified hotspot mutations. (**D**) Frequency distribution plot representing root mean square deviation (RMSD) frequency distribution of the monomer structures during 1 μs molecular dynamic simulation (MDS). (**E**) Frequency distribution plot of radius of gyration showing stable conformation for H2-WT and S305C, and unstable conformation for G309A, S310Y, and P523S. (**F**, **G**) Charts comparing root mean square fluctuations during MDS between H2-WT and domain II mutations (**F**); H2-WT and domain IV mutation (**G**). (**H**) Diagonal cross-correlation matrix analysis showing correlated (blue) and anti-correlated (pink) movements during simulation. (**I**) RMSD frequency distribution plot of respective homodimer structures during 500 ns MDS. (**J**) Line graph representing solvent accessible surface area estimated during simulation for H2-WT and DDMs. (**K**) Graph showing solvation gain on complex formation. Dashed trendlines indicate the trend of ΔG values of H2-WT and its DDMs during simulation.

The prevalent mutations in the dimerization domains of HER2 and HER3 were identified using a comprehensive analysis of multiple tumor tissue databases enlisted in the Materials and Methods section (Fig. 1A). For HER2 dimerization domains, five hotspot mutations were identified, namely S305C, G309A, S310Y, S310F in domain II, and P523S in domain IV. Notably, patients harboring these mutations exhibited significantly reduced overall survival compared to those with wild-type HER2 (H2-WT) (81 vs 185 months; *P*<*0.0001*) (Fig. 1B). To analyze the 2-dimensional structural changes induced by these mutations, three standard tools, Panther,^26^ Provean,^27^ and Mupro,^28^ were employed. All dimerization domain mutants were found to be detrimental to the stability of HER2 structure, except for S305C, which did not destabilize the flanking amino acid chain (Fig. 1C and **Supplementary Table 1**).

Further insights into the 3D structural changes were obtained through long-term molecular dynamic simulation (MDS). Simulation results for H2-WT and S305C mutant revealed stable 3D structures, as evidenced by narrow root mean square deviation (RMSD) distribution (Fig. 1D). In contrast, G309A, S310Y, and P523S structures exhibited high fluctuations, indicated by broader RMSD distribution. Additionally, G309A, S310Y, S310F, and P523S caused drastic conformational changes, as demonstrated by their extensively broad radius of gyration (Rg) distribution (Fig. 1E). Interestingly, some of these point mutations caused a higher degree of instability in the flanking residues compared to H2-WT or S305C, suggesting a potential disruption to conventional HER2:HER2 interactions (Fig. 1F, G). Subsequent diagonal cross-correlation matrix analysis of the top three principal components highlighted that G309A and S310Y mutations compromised the structural integrity of the anking residues in the H2-WT structure (Fig. 1H). A substantial shift from −1.0 to 0.0 in the anti-correlated movement in G309A and S310Y structures further suggested a probable disruption in the binding pocket essential for HER2:HER2 homodimer formation.

To further elucidate the impact of atomic changes induced by HER2 DDMs on their anticipated homodimer formation, we performed docking studies using energy-minimized structures of H2-WT, S305C, G309A, S310Y, S310F, and P523S on the HADDOCK server.^29^ These complexes were then simulated for up to 500 ns each, with the H2-WT homodimer exhibiting the least RMSD fluctuation. Slightly broader RMSD frequency distribution highlighted the marginally less stable nature of the S305C:S305C homodimers. Interestingly, an extended and broad distribution suggested the instability of G309A, S310Y, and P523S homodimers (Fig. 1I). Further insights were obtained by analyzing the solvent accessible surface area (SASA) during the 500 ns simulations. The H2-WT and S305C homodimers showed a decrease of approximately 25 nm^2^ and 21 nm^2^, respectively, suggesting the closure of the dimer interface. In contrast, an increase of approximately 19 nm^2^ and 20 nm^2^ in the SASA for G309A and S310Y homodimers, respectively, suggested unstable dimer formation (Fig. 1J). Conversely, a slight decrease of approximately 5 nm^2^ in SASA for S310F dimer suggested a relatively unstable homodimer. Analyzing the total number of interactions with stabilized dimers revealed an increasing trend for H2-WT and S305C, while G309A and S310Y mutants showed a reverse trend (**Supplementary Fig. S1A**). The major stabilizing bond (hydrogen bond) involved in the stabilization of HER2:HER2 homodimers was comparatively less in number in all mutants studied (**Supplementary Fig. S1B**). Highly negative solvation energy gain on complex formation values (ΔG) during simulation for H2-WT and S305C highlighted the thermodynamic favorability of these homodimers. However, a decreasing trend in the negative values for G309A, S310Y, and S310F indicated thermodynamically unfavorable homodimers for these mutants (Fig. 1K).

### Specific HER2 DDMs drive major change in receptor interaction dynamics from HER2:HER2 to HER2:HER3

**Figure 2:**
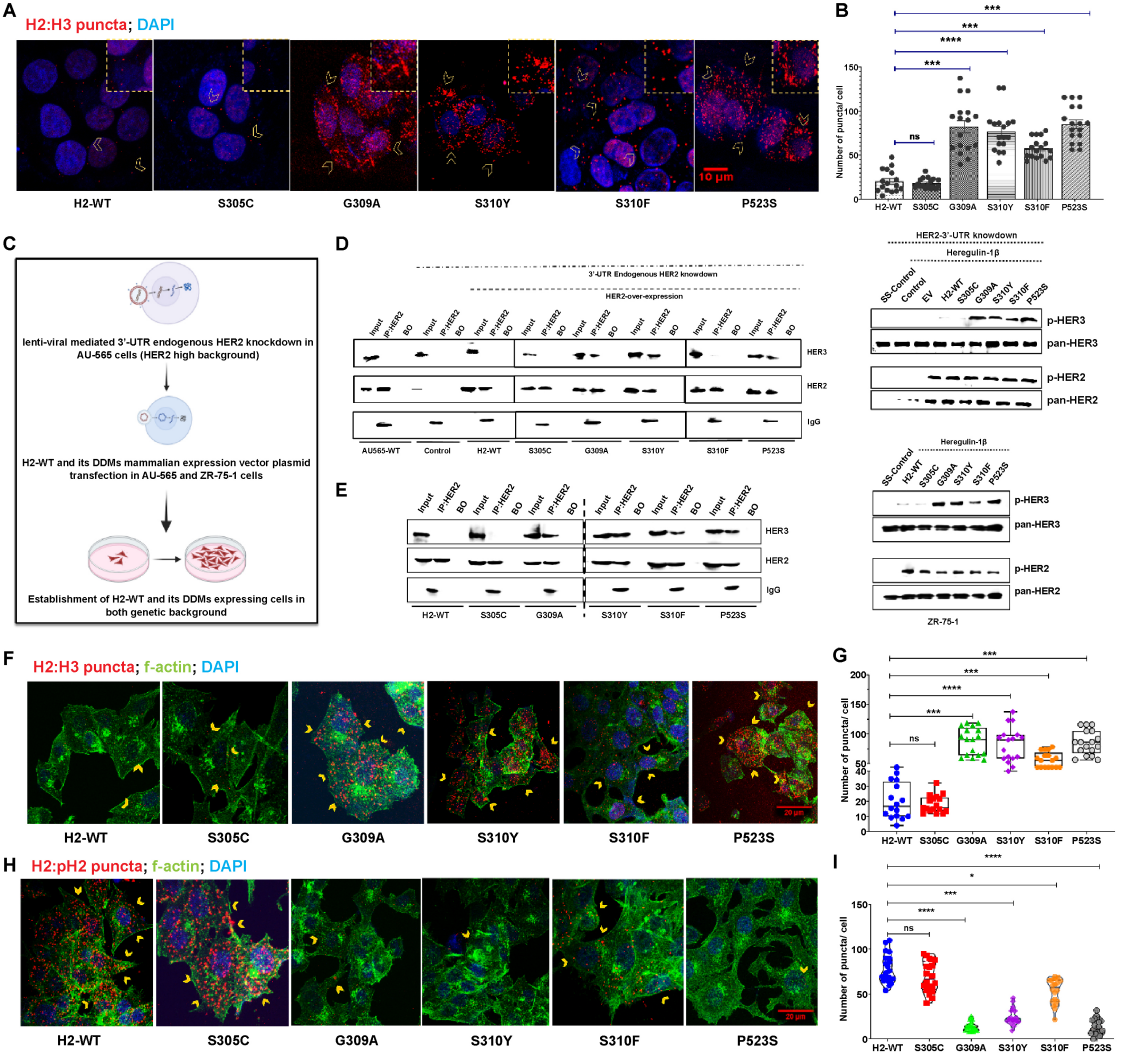
HER2 dimerization domain mutations (DDMs) induce recptor switching in breast cancer cells. (**A**) Representative immunofluorescence photomicrographs of MCF-7 cells overexpressing wild-type HER2 (H2-WT) or DDMs, showing HER2:HER3 PLA puncta (red) after heregulin-1β stimulation. Scale bar: 10 μm. (**B**) Graph showing quantified number of puncta as in (A). (**C**) Schematic depicting the establishment method of H2-WT or DDM expressing cell lines using HER2-high AU-565 and HER2-low ZR-75-1 cells. (**D**) Co-immunoprecipitation result using HER2 mAb in AU-565 cells. BO: bead only control, meaning lysate without HER2 mAb. Side panel showing immunoblot analysis for phospho- and pan-HER2 and HER3 in AU-565 cells. SS-Control: serum starved BC cells, EV: empty pc-DNA3.1 plasmid vector. (**E**) Reverse co-immunoprecipitation (co-IP) using HER3 mAb in ZR-75-1 cells showing physical interaction of H2-WT and DDMs with HER3 in presence of heregulin-1β ligand; side panel shows immunoblot analysis for phospho- and total HER2 and HER3 levels in ZR-75-1 cells. (**F**) Immunofluorescence photomicrographs showing HER2:HER3 PLA puncta (red) in ZR-75-1 cells with stable overexpression of H2-WT or DDMs after heregulin-1β stimulation; cross-stained for F-actin (green) and DAPI (blue); scale bar: 20 μm. (**G**) Graph showing average numbers of puncta counted as in (F). (**H**) PLA assessment results in ZR-75-1 cells for HER2:pHER2 homodimer in the absence of a ligand. (**I**) Graph showing average numbers of puncta counted as in (H). Error bars represent ± standard deviation; *P<0.05; **P<0.01; ***P<0.001, ****P<0.0001; ns- non-significant.

To validate the *in silico* observations, we formulated a hypothesis that HER2 DDMs, particularly G309A, S310Y, and P523S, may induce receptor switching from HER2:HER2 homodimer to HER2:HER3 heterodimer. To test this hypothesis, *in vitro* proximity ligation assay (PLA) was performed using a HER2-low cell line (MCF-7) after transiently overexpressing H2-WT or its DDM expression plasmids. Interestingly, cells expressing G309A, S310Y, S310F, and P523S mutations exhibited a significantly higher number (*P<0.0001*) of HER2:HER3 puncta/cell compared to cells expressing H2-WT or S305C (Fig. 2A, B). These results strongly support the hypothesis that HER2 DDMs, specifically G309A, S310Y, and P523S, induce substantial structural changes in HER2, rendering HER2:HER2 homodimer formation unfeasible. Furthermore, defined secondary structure of proteins analyses revealed disruption in the secondary structures of HER2 during MDS for G309A and S310Y DDMs (**Supplementary Fig. S2A-E**).

Subsequently, to validate the phenotypic effects of the observed receptor switching in BC cells, we established clonal cell lines overexpressing HER2 or one of its DDM transgene by plasmid transfection in ZR-75-1 (another HER2-low cell line). Additionally, in a HER2-high cell line (AU-565), we established a stable endogenous HER2 knockdown clone using lentiviral 3’-UTR transduction, followed by stable overexpression of S305C, S310Y, S310F, and P523S mutation (Fig. 2C). Co-immunoprecipitation (co-IP) results using AU-565 clonal cells clearly showed that G309A, S310Y, and P523S mutations indeed preferred HER3 as a binding partner, whereas H2-WT or the S305C mutation continued to form the homodimers (Fig. 2D). Interestingly, the observed lower amount of bound HER3 in case of S310F mutant suggests that this mutant retains its ability to form the homodimer to some extent. These results were further corroborated by checking phospho-HER3 expression in cells expressing G309A, S310Y, S310F, and P523S after heregulin-1β stimulation. The shift in the interaction dynamics of HER2 due to HER2 DDMs was also validated by performing co-IP and western blot analysis in the BT-474 cell line (another HER2-high BC cell line), (**Supplementary Fig. S3A**). Similarly, reverse co-IP was performed in ZR-75-1 cells stably overexpressing H2-WT or its DDMs, where cells expressing G309A, S310Y, S310F, and P523S mutations showed a strong physical interaction with HER3 in the presence of the ligand (Fig. 2E). However, no detectable interaction with HER3 was observed for H2-WT and S305C expressing cells. A substantial increment in p-HER3 expression further confirmed that G309A, S310Y, S310F, and P523S mutants form stable HER2:HER3 dimers.

PLA was also performed to delineate the HER2:HER2 homodimer or HER2:HER3 heterodimer formation in the presence and absence of the heregulin-1β ligand. Results revealed that ZR-75-1 cells expressing H2-WT and S305C formed approximately 15 and 13 puncta/cell, whereas G309A, S310Y, S310F, and P523S expressing cells exhibited significantly higher number of HER2:HER3 puncta/cells (~86 (*P*<0.001), ~87 (*P*<0.0001), ~65 (*P*<0.001), and ~83 (*P*<0.001), respectively) (Fig. 2F, G). The volume of individual HER2:HER3 puncta was also found to be significantly larger (*P*<0.0001) in the mutant expressing cells (**Supplementary Fig. S3B**). In the absence of the ligand, approximately 77 and 71 HER2:HER2 puncta/cell were observed in H2-WT and S305C overexpressing cells, respectively. Additionally, HER2:HER2 puncta in H2-WT and S305C cells exhibited a significantly larger appearance (*P*<0.0001) than the cells expressing other mutants (Fig. 2H, I and **Supplementary Fig. S3C**). Similarly, in engineered 3’UTR endogenous knockdown AU-565 cells overexpressing wild-type or DDMs, higher numbers of HER2:HER3 puncta were observed in S310Y, S310F, and P523S expressing cells as compared to H2-WT and S305C expressing cells (**Supplementary Fig. S3D**).

### HER2 DDMs induce alterations in receptor dimerization, leading to downstream signaling switch

**Figure 3:**
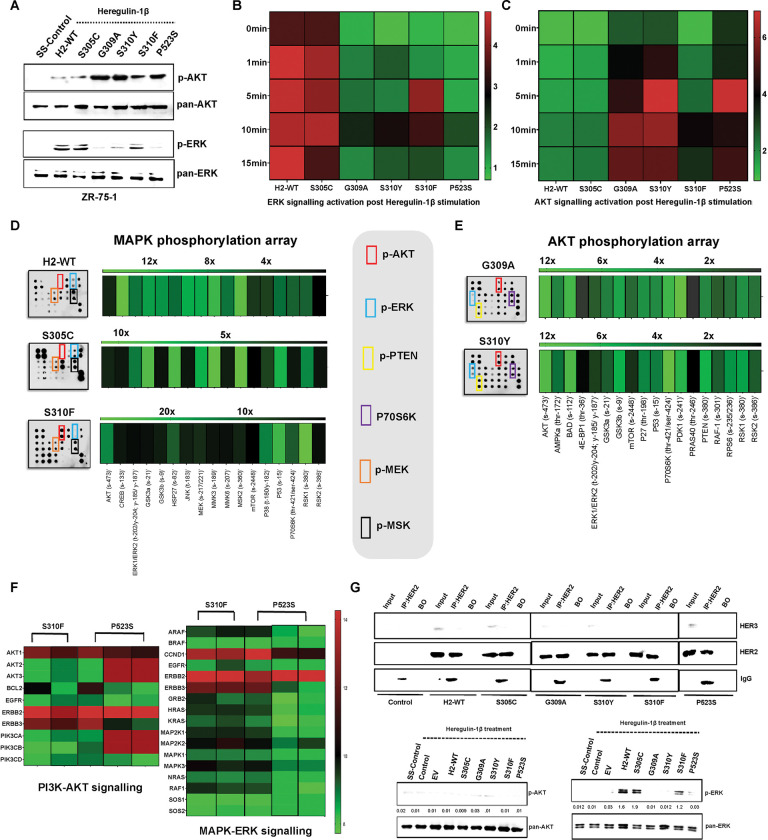
Recptor switching due to HER2 dimerization domain mutations (DDMs) in breast cancer cells changes downstream signaling. (**A**) Western blot showing the levels of p-AKT and p-ERK in ZR-75-1 breast cancer cells expressing HER2 and its stable DDMs. (**B**, **C**) Heatmap showing ERK activation measured using p-ERK bioluminescence resonance energy transfer (BRET) sensor (**B**) and AKT activation measured using p-AKT BRET sensor (**C**) after heregulin-1β stimulation in ZR-75-1 cells overexpressing wild-type HER2 (H2-WT) and DDMs. (**D**) Representative array and heatmap showing the phosphorylation of proteins involved in MAPK signaling cascade in H2-WT, S305C, and S310F mutation expressing cells, as well as in (**E**) AKT signaling cascade in G309A and S310Y mutation expressing cells. (**F**) Heatmap showing the corresponding transcript level measured for MAPK and AKT signaling molecules in H2-WT and its DDMs harboring patients with breast cancer; each column represents a patient from METABRIC dataset. (**G**) Co-immunoprecipitation result using HER2 mAb showing physical interaction specificity of HER2 DDMs with HER3 after HER3-TRIM-ing in ZR-75-1 clonal cells as marked. Loading amount was one-fifth of the amount used for immunoprecipitation; BO: bead only. Bottom immunoblot panels showing levels of phospho- and total AKT and ERK in H2-WT and its DDMs expressing cells after ligand stimulation and HER3-TRIM-ing; Control-SS: serum starved ZR-75-1 cells, EV: pcDNA-3.1 empty vector.

Transient over-expression of H2-WT and its DDMs in both HER2-low (ZR-75-1 and MCF-7) and HER2-high (AU-565 and BT-474) cell lines revealed a notable receptor switch from HER2:HER2 to HER2:HER3 interaction in cells expressing G309A, S310Y, and P523S mutations. This altered interaction dynamics was found to induce a downstream signaling switch. Furthermore, ZR-75-1 and AU-565 cells with stable overexpression of G309A, S310Y, and P523S mutants exhibited high p-AKT levels after ligand stimulation. In contrast, p-ERK levels were high in cells expressing H2-WT and S305C mutation (Fig. 3A and **Supplementary Fig. S4A**). The cells expressing S310F mutant, which formed both HER2:HER2 and HER2:HER3 dimer, exhibited evident activation of both ERK and AKT in immunoblot analysis.

Furthermore, we evaluated p-AKT and p-ERK status by utilizing bioluminescence resonance energy transfer (BRET) phospho-sensors previously developed by our group for assessing ERK and AKT activation and signaling switch induced by HER2 receptor switching.^30^ Upon heregulin-1β stimulation (10 ng/mL), H2-WT and S305C mutant expressing cells exhibited no significant increase in the p-ERK BRET sensor. Notably, G309A, S310Y, and P523S expressing cells also did not reveal a dramatic increase in the p-ERK BRET readings (Fig. 3B). Despite no substantial increment in the p-ERK sensor readings, H2-WT and S305C expressing cells showed an already elevated BRET ratio of p-ERK sensor compared to other mutants. These results suggest that ERK signaling is activated by HER2 homodimer in a ligand-independent manner. In contrast, following heregulin-1β stimulation p-AKT sensor revealed a significant enhancement in AKT signaling in G309A, S310Y, and P523S mutants by approximately 6-fold, 6.9-fold, and 7-fold, respectively (Fig. 3C). Interestingly, a marginal increase in p-AKT readings in H2-WT and S305C expressing cells post-ligand stimulation suggests a preference for ERK signaling activation in these cells. S310F-harboring cells exhibited approximately 4-fold activation in ERK signaling and *4.5-fold* activation in AKT signaling post-ligand treatment. Furthermore, the p-AKT and p-ERK sensors were employed to evaluate the effect of trastuzumab treatment, which specifically inhibits HER2:HER2 homodimer mediated signaling on H2-WT and its DDMs-expressing cells. Trastuzumab treatment demonstrated dose-dependent inhibition in p-AKT and p-ERK signaling in H2-WT expressing cells (*P*<0.0001; *P*<0.001, respectively). In contrast, G309A, S310Y, and P523S expressing cells exhibited intrinsic resistance to trastuzumab treatment (**Supplementary Fig. S4B, C**). In S310F mutant expressing cells, trastuzumab treatment inhibited AKT (*P*<0.001) and ERK (*P*<0.01) signaling, although the extent was not as substantial as that observed in H2-WT and S305C expressing cells.

To provide further evidence on the differential activation status of the downstream signaling cascade in ZR-75-1 cells expressing HER2 and its DDMs, human AKT and MAPK pathway phosphorylation arrays were employed. Notably, the MAPK array revealed an approximately 8-fold and 7-fold increase in p-ERK levels in H2-WT and S305C expressing cells. In addition, an ~11-fold and ~6-fold augmentations were observed in upstream p-MEK and a ~10-fold increase in downstream p-MSK levels in H2-WT and S305C cells, indicating the complete activation of the MAPK signaling (Fig. 3D). In contrast, the AKT array showed ~12-fold increase in p-AKT levels in G309A and S310Y expressing cells, respectively. Moreover, a ~5-fold and ~6-fold increase in the negative regulator PTEN S380 phosphorylation and ~7-fold and ~12-fold increase in downstream P70S6K highlighted functional AKT signaling cascade in G309A and S310Y expressing cells, respectively (Fig. 3E). Intriguingly, S310F expressing cells showed ~17-fold higher p-AKT, ~3-fold higher p-ERK, ~11-fold higher P70S6K, and ~7-fold higher p-MSK levels, suggesting the activation of both ERK and AKT signaling pathways.

To strengthen our understanding of the signaling switch induced by HER2 DDMs, transcriptome, and protein expression datasets from patients with BC in the METABRIC and Firehose Legacy cohorts, respectively, were analyzed. Notably, in METABRIC, similar to H2-WT (data not presented), patients harboring S305C mutation exhibited high expression of genes associated with the ERK signaling, while patients harboring S310F, G309A, and P523S mutations displayed elevated expression levels of genes associated with AKT signaling (Fig. 3F). Moreover, Firehose Legacy dataset showed similar trend suggesting that these mutations preferentially interact with HER3, leading to the activation of PI3K-AKT signaling cascade (**Supplementary Fig. S4D**). To validate the shift in HER2 interaction dynamics following G309A, S310Y, S310F, and P523S mutations, we employed the ‘TRIM-ing’ mediated direct protein knock down methodology. Here, endogenous total HER3 protein was removed to confirm the specificity of HER2:HER3 heterodimer formation in G309A, S310Y, and P523S mutant HER2-expressing cells. Results revealed that under the HER3-TRIM-ing condition, heregulin-1β stimulation failed to induce physical interaction with HER3 in these mutant expressing cells. Moreover, a dramatic decrease in p-AKT levels was observed in G309A, S310Y, P523S, and S310F expressing cells, establishing the specific link between HER2:HER3 interaction and subsequent AKT signal activation. Additionally, this experiment provided evidence that H2-WT and S305C expressing cells continued to form HER2:HER2 homodimer, with no observed change in p-ERK levels (Fig. 3G).

### Interaction with HER3 in cells expressing HER2 DDMs trigger intrinsic resistance to HER2-targeted therapy

**Figure 4:**
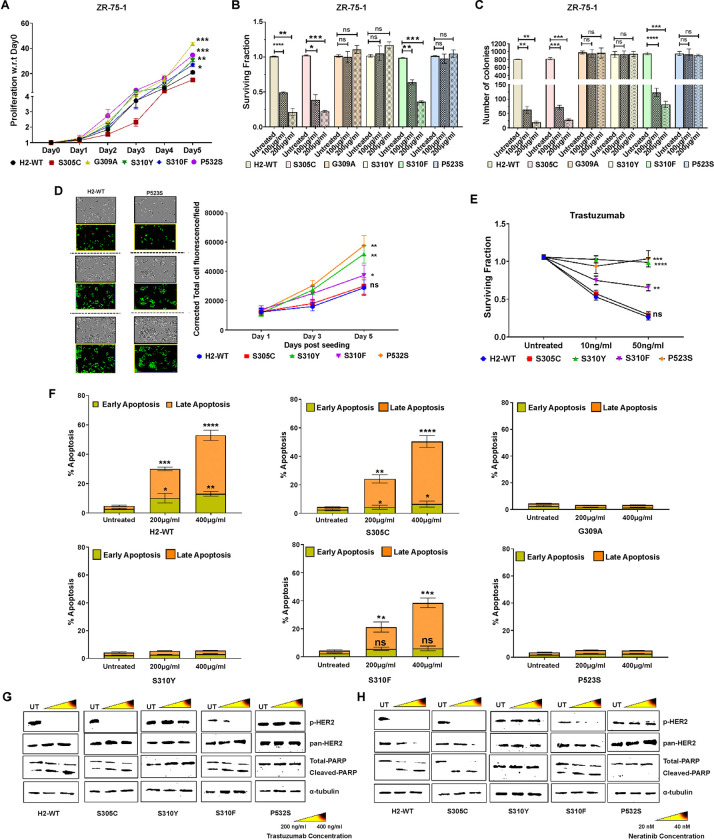
Breast cancer cells expressing G309A, S310Y, and P523S induce intrinsic resistance to HER2-personalized medicines. (**A**) Graph showing proliferation rate measured at every 24 h for 5 days in ZR-75-1 cells expressing wild-type HER2 (H2-WT) and its dimerization domain mutations (DDMs). (**B**, **E**) Graphs showing the long-term clonogenic survival potential of H2-WT and its DDMs expressing ZR-5-1 cells (**B**) and AU-565 cells (**E**) in the presence or absence of trastuzumab. (**C**) Graph showing the 3D anchorage-independent growth of ZR-75-1 cells expressing HER2-WT and its DDMs in the presence of trastuzumab. (**D**) Graph showing proliferation of H2-WT and its DDMs expressing cells. Representative photomicrographs and green fluorescence images depict proliferation of H2-WT and P523S expressing AU-565 cells at days 1, 3, and 5. (**F**) Graphs showing percentage of early and late apoptosis in ZR-75-1 cells expressing H2-WT or other DDM studied after trastuzumab treatment. (**G**, **H**) Western blots of p-HER2, pan-HER2, cleaved PARP, and α-tubulin performed after treatment with high doses of trastuzumab (G) and neratinib (H) using AU-565 cells stably expressing H2-WT or DDMs. Error bars for all experiments represent ± standard deviation; *P<0.05; **P<0.01; ***P<0.001 ****P<0.0001; ns- non-significant.

Proliferation assay of ZR-75-1 cells expressing HER2 mutants revealed that G309A (*P*<0.001), S310Y (*P*<0.01), and P523S (*P*<0.001) expressing BC cells were significantly more proliferative than H2-WT and S305C expressing BC cells, suggesting that BC cells harboring these mutations may be more aggressive in nature (Fig. 4A). Furthermore, short-term cell survival assessment revealed that mutants interacting with HER3 (G309A, S310Y, and P523S expressing cells) were resistant to trastuzumab (first-line of therapy) and neratinib (last line of therapy). However, H2-WT and S305C expressing cells remained highly sensitive to trastuzumab (*P*<*0.0001*; *P*<*0.001*) or neratinib (*P*<*0.0001*; *P*<*0.001*) (**Supplementary Fig. S5A**). Corroborating these results, G309A, S310Y, and P523S expressing BC cells were non-responsive to these drugs in a long-term survival assessment. Conversely, H2-WT expressing cells exhibited high sensitivity to trastuzumab (*P*<*0.01*) and neratinib (*P*<*0.001*). Similarly, S305C was highly responsive to trastuzumab (*P*<*0.001*) and neratinib (*P*<*0.01*). S310F expressing cells were slightly less sensitive to trastuzumab (*P*<0.01) and neratinib (P<0.01) than H2-WT and S305C expressing cells (Fig. 4B and **Supplementary Fig. S5B**). In the soft agar assay, G309A, S310Y, and P523S mutants were also resistant to trastuzumab treatment (Fig. 4C).

Similar results were found in AU-565 HER2 mutants over-expressing cells, wherein S310Y (*P*<*0.01*) and P523S (*P*<*0.01*) expressing cells demonstrated aggressive growth compared to H2-WT and S305C expressing cells (Fig. 4D). AU-565-S310Y (*P*<*0.0001*; *P*<*0.0001*) and AU-565-P523S (*P*<*0.001*; *P*<*0.0001*) cells exhibited resistance to both trastuzumab and neratinib, respectively, when compared to AU-565-H2-WT and AU-565-S305C expressing cells (Fig. 4E and **Supplementary Fig. S5C**). Annexin-propidium iodide (PI) assessment of ZR-75-1 cells expressing H2-WT and its DDMs underscored the resistance of G309A, S310Y, and P523S expressing cells even at elevated doses of trastuzumab (200 μg/mL and 400 μg/mL) (Fig. 4F and **Supplementary Fig. S5D**). Together, these results suggest that due to HER2:HER3 heterodimer formation, these cells have lost dependence on HER2:HER2 signaling. Validating these findings, we also evaluated cleaved PARP levels in AU-565-H2-WT and some of the DDM expressing cells following treatment with trastuzumab (200 ng/mL and 400 ng/mL) and neratinib (20 nM and 40 nM). A pronounced increase in cleaved PARP levels was observed in H2-WT and S305C expressing cells in a dose-dependent manner, indicating efficient cellular apoptosis. However, S310Y and P523S expressing cells exhibited resistance to both the HER2-targeted drugs (Fig. 4G, H).

**Figure 5:**
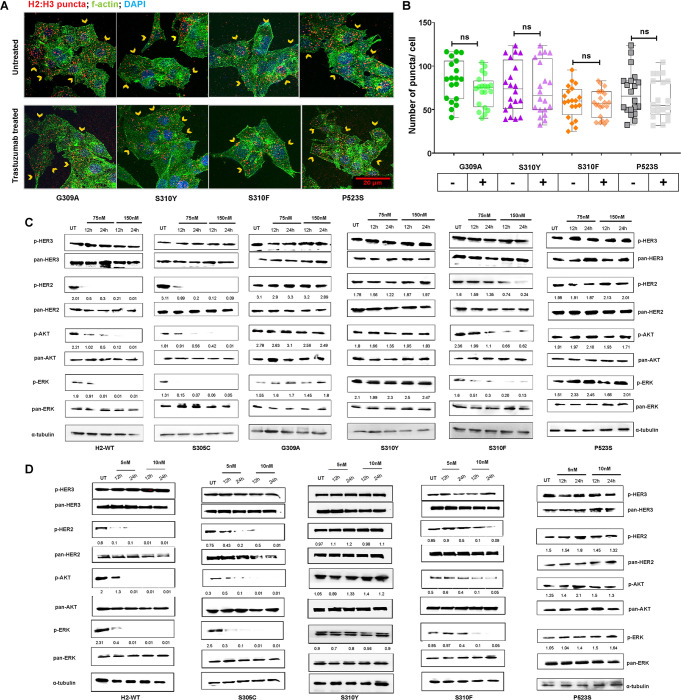
Trastuzumab and neratinib were ineffective in cells expressing HER2 dimerization domain mutations (DDMs). (**A**) Immunofluorescence images comparing HER2:HER3 PLA puncta (red) after trastuzumab treatment (300 μg/mL) for 24 h in ZR-75-1 cells stably expressing G309A, S310Y, S310F or P523S mutation. Scale bar: 20 μm (**B**) Graph showing the number of puncta counted from images as in (**A**); ‘+/−’ represent trastuzumab treatment. Error bars represent ± standard deviation; ns- non-significant. (**C**) Western blots showing levels of phospho- and pan-HER2, HER3, AKT, and ERK, along with α-tubulin loading control in ZR-75-1 cells after neratinib treatment. (**D**) Immunoblots showing levels of phospho- and pan-HER2, HER3, AKT, and ERK, along with α-tubulin loading control in AU-565 cells expressing H2-WT, S305C, S310Y, S310F and P523S, after treatment of different doses of neratinib for 12 and 24 h. Pan-blots (total) are used for quantification of the respective phospho-protein in both C and D.

To compare HER2:HER3 interaction before and after trastuzumab treatment, PLA analysis was performed, which showed non-significant changes in red puncta formation in cells expressing DDMs. These results provide further evidence to our previous findings showing G309A, S310Y, and P523S are intrinsically resistant to trastuzumab, whereas S310F expressing cells formed fewer (~55 puncta/cell) puncta (Fig. 5A, B). To unravel the underlying mechanism of reduced drug sensitivity in the DDM expressing cells under neratinib treatment, we performed immunoblot analysis across all lines developed using ZR-75-1 and AU-565 cells. Upon neratinib treatment, cells showed substantial attenuation in the p-HER2, p-ERK and p-AKT expressions in H2-WT and S305C expressing ZR-75-1 cells, and a slight attenuation in S310F cells. Meanwhile, G309A, S310Y, and P523S expressing cells showed no change in expression for the above activation marker, confirming the intrinsic resistant nature of these cells (Fig. 5C). To further validate, H2-WT or DDM expressing AU-565 cells were also treated with neratinib for 12 h and 24 h. These results revealed similar information about neratinib drug sensitivity of H2-WT and S305C expressing cells, a moderate sensitivity of S310F but an intrinsic resistance phenotype of S310Y and P523S cells, emphasizing inefficient neratinib inhibitory effect when signaling operates through HER2:HER3 hetero-dimer (Fig. 5D).

### BC tumors expressing G309A, S310Y and P523S DDMs are insensitive to HER2-targeted therapeutics

**Figure 6:**
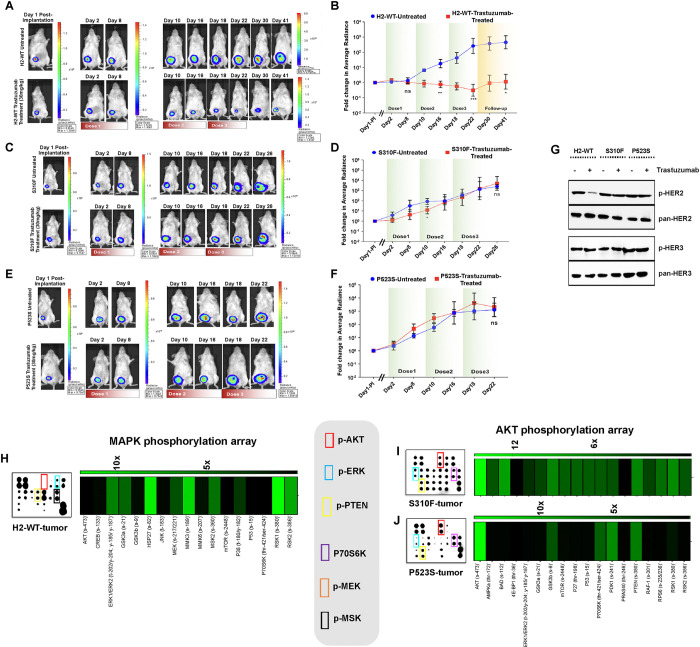
Analysis of *in vivo* therapeutic response of trastuzumab in orthotropic breast tumors. (**A**) Representative bioluminescence images of H2-WT tumor-bearing mice in untreated and treated arms. (**B**) Graph representing quantitative photonic signal (average radiance) from tumor site of the mice over time. (**C**) Representative bioluminescence images of S310F tumor-bearing mice in untreated and treated arms. (**D**) Graph representing quantitative photonic signal (average radiance) from tumor site of the mice over time. (**E**) Representative bioluminescence images of P523S tumor-bearing mice in untreated and treated arms. (a) Graph representing quantitative photonic signal (average radiance) from tumor site of the mice over time. Error bars represent ± standard deviation for five mice/group. (**G**) Western blots showing levels of p-HER2, pan-HER2, p-HER3, and pan-HER3 measured from harvested H2-WT, S310F, and P523S tumor samples treated or untreated with trastuzumab. (**H**, **I**, **J**) Heatmaps showing the levels of ERK signaling cascade in H2-WT, and AKT signaling in S310F and P523S tumors.

For preclinical evaluation, we used previously developed firefly luciferase-labeled ZR-75 cells overexpressing H2-WT, S310F, and P523S to assess the *in vivo* tumor growth and trastuzumab treatment efficacy (**Supplementary Fig. S6A**). H2-WT over-expressing cells showed a remarkable ~10^4^-fold (*P*<*0.001*) decrease in average radiance values after receiving three weekly doses of trastuzumab (30 mg/kg intra-peritoneal injection per week), indicating substantial tumor growth reduction. Interestingly, tumor volume regression persisted even after treatment cessation, suggesting the efficacy of trastuzumab in tumor regression (Fig. 6A, B, **Supplementary Fig. S6B**). The S310F over-expressing BC tumor responded to the first dose of trastuzumab as indicated by ~10-fold decrease in average radiance values. However, compared to H2-WT expressing cells, these cells depicted lesser sensitivity over time (Fig. 6C, D, **Supplementary Fig. S6B**). In contrast, P523S expressing BC cells displayed intrinsic resistance to trastuzumab therapy, as no significant change in bioluminescence (BLI) signal was observed in average radiance values between the untreated and trastuzumab-treated groups (Fig. 6E, F, **Supplementary Fig. S6B**). Notably, P523S and S310F expressing cells exhibited significant aggressiveness compared to H2-WT expressing cells. Post-treatment immunoblot analysis of the tumors revealed trastuzumab-induced dramatic reduction in p-HER2 levels only in the H2-WT tumor group, with no effect observed in S310F and P523S tumor groups (Fig. 6G). Subsequently, MAPK array analysis of H2-WT tumors revealed ~6-fold, ~5-fold, and ~6-fold increase in p-ERK, p-MEK, and p-RSK levels, respectively, indicating major cascade activation of ERK signaling pathway (Fig. 6H). AKT array analysis of S310F tumors showed ~13-fold, ~5-fold, ~5-fold increase in p-AKT, p-ERK, P70S6K levels, respectively, and ~6-fold increase in p-PTEN levels, suggesting activation of both AKT and ERK signaling pathways (Fig. 6I). Furthermore, ~15-fold and ~8-fold increases in p-AKT and P70S6K levels in P523S tumors, respectively, suggest a signaling switch from ERK to AKT due to HER2:HER3 heterodimer formation (Fig. 6J).

### HER2 DDMs interacting with HER3 are more aggressive in nature but sensitive to tucatinib

**Figure 7:**
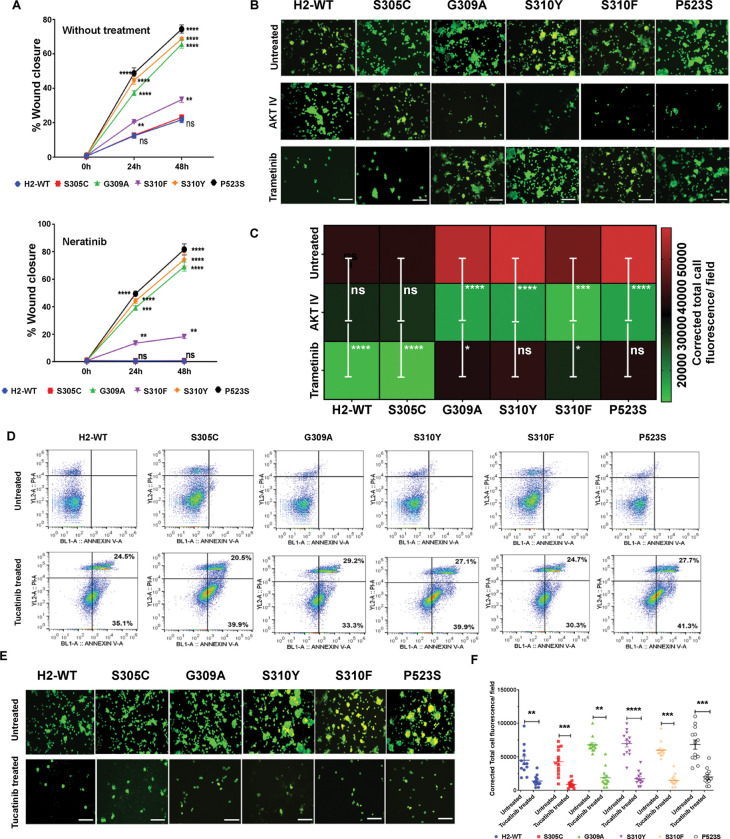
Effect of HER2 dimerization domain mutations (DDMs) on cell invasion and migration properties. (**A**) Graph showing collective migratory capabilities of wild-type HER2 (H2-WT) or DDM expressing ZR-75-1 cells. G309A, S310Y, and P523S mutant expressing cells exhibit nearly complete wound closure within 48 h, indicating enhanced migratory potential. These cells remain unresponsive to neratinib treatment, showing no change in wound closure capabilities. (**B**) Representative photomicrographs of CalceinAM stained H2-WT or DDM expressing ZR-75-1 cells showing matrigel invasive capabilities in absence or presence of p-AKT and p-ERK inhibitors. (**C**) Heatmap showing the quantified total cell fluorescence/field of results as in B. Corrected total cell fluorescence values were compared between untreated and treated conditions, ns-non-significant; **P<0.01; ***P<0.001 ****P<0.0001. (**D**) Annexin V/PI-stained ow cytometry results showing H2-WT or the DDMs expressing cells before and after tucatinib treatment. (**E**) Representative photomicrographs showing matrigel invasive capabilities of H2-WT or the DDM expressing cells with or without tucatinib treatment. (**F**) Graph showing the quanti ed total cell fluorescence/field of results as in E. Error bars for all experiments represent ± standard deviation; **P<0.01; ***P<0.001 ****P<0.0001.

Results accumulated so far suggest that HER2:HER3 heterodimer leading to AKT signaling drives higher proliferation in G309A, S310Y, S310F, and P523S mutant harboring BC cells. To evaluate whether these cells possess altered metastatic phenotypes, we assessed their invasion and collective migration abilities. H2-WT and S305C expressing ZR-75-1 cells exhibited ~20% wound closure, which was dramatically inhibited after trastuzumab and neratinib treatment (Fig 7A and **Supplementary Fig. S7A-B**). Interestingly, G309A, S310Y, and P523S expressing cells healed wounds at a much faster rate within the same time, i.e., ~61% (P<0.0001), ~65% (P<0.0001), and ~70% (P<0.0001) respectively. S310F overexpressing cells demonstrated ~10% (P<0.01) higher migratory capacity than H2-WT cells. Treatment with HER2-targeted regimen led to a slight drop in the migratory capabilities of these cells. We also explored the impact of ERK or AKT inhibition on invasiveness using respective inhibitors on ZR-75-1-HER2 DDMs expressing cells. H2-WT and S305C mutant expressing cells exhibited dependence on HER2:HER2 homodimer regulated ERK signaling, resulting in a significant decrease of invasive potential for both H2-WT and S305C cells (P<0.0001) upon ERK inhibition (Fig. 7B, C). Validating the signaling switch from ERK to AKT in G309A, S310Y, and P523S cells due to HER2:HER3 heterodimer prevalence, we found that only AKT inhibition caused ~10-fold (P<0.0001), ~12-fold (P<0.0001), and ~15-fold (P<0.0001) decrease in the invasive potential, respectively (Fig. 7B, C). Notably, the invasiveness of S310F mutant harboring cells was also diminished with ERK and AKT inhibition. However, significant inhibition was observed with AKT inhibition, suggesting that although these cells exhibit activation of both ERK and AKT signaling pathways, their dependence primarily lies in AKT signaling mediated by HER2:HER3 heterodimer.

Further, we assessed *in vivo* metastasis in orthotopic tumor-bearing mice using BLI imaging as detailed in the previous section. In H2-WT expressing tumor-bearing mice, we observed 100% metastasis in lungs and bone, 80% in liver, and 40% in the brain. Notably, trastuzumab treatment significantly reduced metastasis to the lungs (P<0.01), liver (P<0.05), bone (P<0.01), and brain (P<0.05) (**Supplementary Fig. S7C, D**). Interestingly, S310F mutant bearing tumor exhibited augmented metastatic incidence to all these sites compared to H2-WT group. Trastuzumab treatment significantly decreased the average radiance of lung (*P*<*0.05*) and liver (*P*<*0.05*) metastases, and dramatically reduced bone and brain metastasis. Consistent with our earlier observations, P523S mutant expressing cells demonstrated the highest metastatic loads in all major distant organs among the tested groups.

Recently, the Food and Drug Administration (FDA) approved tucatinib for use in metastatic HER2+ BC. A report highlighted the efficacy of tucatinib in inhibiting signaling pathways driven by both HER2:HER3 and HER2:HER2 interactions.^31^ Short-term cell viability assessment indicated that tucatinib significantly inhibits the proliferation of H2-WT or the DDM expressing cells with comparable IC_50_ [IC_50_ range: 5.8–aa23.5 nM] (**Supplementary Fig. S8A, B**). Treatment with escalating doses of tucatinib significantly reduced the clonogenic potential of H2-WT and all mutants expressing cells (P<0.0001) (**Supplementary Fig. S8C**). Apoptosis assessment after tucatinib treatment in these cell lines revealed a notable increase from ~1.9 ± 1.2% to ~36.63 ± 4.39 % in early apoptotic cells, as well as late apoptotic cells from 2.3 ± 0.5% to ~25.62 ± 3.09 %, confirming the efficacy of tucatinib in inhibiting both cases where HER2:HER2 homodimer and HER2:HER3 heterodimer signaling drives the cellular phenotype (Fig. 7D). The disruption of driver signaling after tucatinib treatment led to ~*6.5-fold* (*P*<*0.01*), ~*7.9-fold* (*P*<0.001), *and* ~*8.2-fold* (*P*<0.001) decrease in the invasive potential of G309A, S310Y, and P523S expressing cells, respectively (Fig. 7E, F). Interestingly, the average cell migratory potential of G309A, S310Y, S310F, and P523S expressing cells was reduced from ~85% ± 7.8% to ~3% ± 0.08% (P<0.0001) after tucatinib treatment (**Supplementary Fig. S8D, E**).

## Discussion

Successful treatment of patients with BC using personalized medicines that lead to improved clinical outcomes is now a well-established fact. Tamoxifen plus fulvestrant for ER-positive BCs and trastuzumab for HER2-positive BC cases is noteworthy as first-line therapy.^17^ The crucial role of ‘HER2 oncogenic addiction’ in cancer cells underscores the treatment’s efficacy, where downstream signaling through HER2 drives the BC cell proliferation and growth. Blocking HER2 signaling, either through a monoclonal antibody (trastuzumab) or a small molecule inhibitor (neratinib), effectively inhibits cancer growth. However, patients developing resistance to these therapeutics are well evidenced in the literature, either due to compensatory pathway activation or mutations hindering the binding of small molecule inhibitors to the tyrosine kinase domain. To the best of our knowledge, this study is the first to explore various pathogenic mutations presented in patients on the HER2 interaction domains, aiming to elucidate their impact on interaction dynamics and, consequently, dictating the therapeutic response to the personalized medicines in BC cells.

Analyzing the TCGA data, we have identified and evaluated five hotspot mutations, namely, S305C, G309A, S310F, S310Y in domain II, and P523S in domain IV. Patient data suggested that these mutations are prevalent and affect the overall survival of patients with HER2-positive BC. Simulation analyses of H2-WT and its mutant dimer structures revealed no fluctuation during the simulations, indicating stable HER2:HER2 interactions for H2-WT and S305C homodimers. The previously studied S310F mutation,^3^ exhibited a somewhat unstable interaction, while G309A, S310Y, and P523S showed notably unstable interactions when compared to H2-WT. Interestingly, the S310F mutant formed homodimers; however, the stability was lower than that of H2-WT. Therefore, based on new evidence gathered for the first time, the presence of G309A, S310Y in domain II or P523S mutation in domain IV in patients undergoing HER2 personalized treatment is expected to represent significant alterations in HER2 receptor structure. Although domain IV is known to stabilize HER2 interactions mediated by domain II in the presence or absence of ligands,^32^ our analysis of the single domain IV mutation (P523S) revealed substantial fluctuations in the anking region of domain IV, suggesting a pivotal role played by this domain during receptor interaction.

Following substantial insights gained from *in silico* analyses on decreased stability caused by HER2 DDMs, we conducted co-IP experiments with HER2 mutants in both HER2-low (MCF-7 and ZR-75-1) and HER2-high (BT-474 and AU-565) cell lines. Interestingly, G309A, S310Y, and P523S mutants preferred HER3 as their binding partner rather than HER2, whereas H2-WT and S305C mutants continued to favor the HER2:HER2 homodimers. The S310F mutant interacted with both HER2 and HER3 in the presence of heregulin-1β. We subsequently generated stable cells expressing either H2-WT or its DDMs in ZR-75-1 and AU-565 cells and performed PLA to cross-verify co-IP results. The results indicated that G309A, S310Y, S310F, and P523S cells form clusters of HER2:HER3 interactions in BC cells. These novel findings are consistent with the reports suggesting the accumulation of HER2 receptors in specialized membrane regions.^11,33^ Furthermore, cross-validating conventional HER2:HER2 homodimer signaling revealed that PLA clusters exclusively in H2-WT and S305C.^33,34^ Subsequently, we focused on understanding how the altered receptor interaction in cells bearing the mutant forms of HER2 may influence the downstream signaling activation by employing previously validated live cell phospho-BRET reporter sensors for ERK and AKT activation. These BRET assays were designed for precise, biophysical distance-dependent measurements, providing confirmatory results of phospho-activation from live cells.^30,35^ The results suggested a clear signaling shift from MAPK activation in wild-type or S305C mutant cells to AKT activation in G309A, S310Y, and P523S mutant cases. Subsequently, the human MAPK and AKT kinase array identified the involvement of relevant kinases, thus confirming the signaling switch from ERK in H2-WT or S305C expressing cells to the PI3K-AKT in G309A, S310Y, or P523S mutant bearing cells. Therefore, our observed results affirm the switching of distinct arms of growth-promoting signals in HER2 positive BC cells reported before in drug resistant cases.^1,12,36^

We further evaluated the potential impact on therapeutic efficacy to HER2 personalized medicines and observed that while H2-WT and S305C expressing cells are sensitive to trastuzumab treatment, the S310F mutant cells demonstrated reduced sensitivity, with the G309A, S310Y, and P523S expressing cells being resistant. Thereafter, using direct knockdown of HER3 target by TRIM-ing method, without compensatory activation of other EGFR family members present in the cell, our results confirmed that G309A, S310Y, and P523S mutant bearing cells indeed drive cellular oncogenesis via AKT activation.^37^ Furthermore, BC cells expressing G309A, S310Y, and P523S mutants exhibited higher proliferation than H2-WT, S305C, and S310F expressing cells did, suggesting their potential aggressive phenotype. This may be attributed to p95-HER2 and HER3 driven signaling, as reported previously for association with aggressive HER2-positive BC.^38,39^ We also confirmed the drug resistant nature of HER2 DDMs in vitro and *in vivo* using orthotopic mouse model. The P523S mutant expressing tumors showed non-responsiveness to trastuzumab treatment, whereas S310F expressing tumors showed partial response to the drug. Furthermore, thorough *in vitro* assessments on G309A, S310Y, and P523S expressing cells demonstrated increased invasiveness and migratory capabilities as compared to H2-WT and S305C cells. Similarly, preclinical assessment of distant metastasis suggests that P523S-expressing tumors represent the most aggressive type, compared to H2-WT or S310F expressers. Targeted inhibition of AKT by AKT-IV significantly deceased the invasive capabilities of G309A, S310Y, and P523S expressing cells. In contrast, H2-WT and S305C expressing cells demonstrated sensitivity to ERK inhibition tested using trametinib (MEK inhibitor). These results provide evidences that in HER2+ BC, cells harboring G309A, S310Y, and P523S mutants are functionally dependent on AKT signaling, while H2-WT and S305C mutant expressers are functionally dependent of ERK signaling.

Based on the HER2CLIMB trial results on metastatic HER2-positive BC, tucatinib has recently received FDA approval for use. It has been reported to effectively inhibit HER2:HER3 heterodimer mediated signaling.^31,40^ Consistent with these reports, our findings indicate that tucatinib can restrict the proliferation, invasiveness, and collective migratory potential of cells expressing H2-WT or the DDMs tested here in a dose-dependent manner. Therefore, results from our study add evidence that tucatinib’s coverage for observed drug efficacy is potentially due to its ability to disrupt both HER2:HER2 and HER2:HER3 driven signaling axis.

These results highlight severe therapeutic consequences of some of the hotspot DDMs present in HER2 positive BC cases. Nevertheless, this study has a few limitations, including the difficulty of addressing tumor heterogeneity in patients harboring mutation in an *in vitro* experimental setting. Furthermore, to understand the therapeutic implications *in vivo*, patient-derived xenografts (PDX) would have been ideal; however, currently, the available BC-PDX are primarily from ER+ tumor subtypes. Moreover, considering the clinical utility of antibody-drug conjugates, such as T-DM1 and T-DXd, future studies may screen for responsiveness to these drugs in pathogenic DDM conditions.

In conclusion, HER2 homodimerization mediated by domain II and IV is found pivotal in driving oncogenic addiction in HER2-high BC subtype. Our study reveals novel information on deleterious effects of multiple pathogenic mutations present in HER2 dimerization domain structure. The presence of these mutations, particularly G309A, S310Y, and P523S, can cause severe disruption in HER2:HER2 homodimer formation, thereby favoring the switch to the formation of HER2:HER3 heterodimer. This receptor switching induces significant changes in downstream signaling activation, shifting from MAPK to AKT signaling. Moreover, we demonstrated that these mutations exhibit aggressiveness and resistance to drugs such as trastuzumab and neratinib. Considering the American Society of Clinical Oncology recommendations that typically base trastuzumab therapy on IHC and FISH score values, our findings suggest that patients harboring the reported HER2 DDMs may not respond to the currently approved first- or second-line personalized therapies. Approximately 3% of HER2-positive patients harboring G309A, S310Y, and P523S mutations may not respond to trastuzumab (first-line) or neratinib (last-line) treatments, as both drugs are ineffective in inhibiting HER3 signaling. Our early results bring fresh evidence that tucatinib could be a more effective therapeutic option for BC cases harboring these majorly deleterious HER2 DDMs.

## STAR Methods

### Experimental Model and Study Participant Details

#### Establishment of stable cell line

ZR-75-1 and AU-565 breast cancer cell lines were maintained in ATCC recommended RPMI-1640 and ATCC-RPMI-1640 high glucose, respectively, completed with 10% FBS and 1% P/S. Transfection of H2-WT or the DDMs plasmid expression vectors were done using Lipofectamine 2000 reagent (Thermo Fisher Scientific, USA).

### Method Details

#### Materials

Trastuzumab (Vivitra^™^), developed by Zydus Lifesciences (Ahmedabad, India), was used in this study. Neratinib (# S2150), tucatinib (# S8362), trametinib (#S2673), and AKT-IV (#E2384) were purchased from Selleck Chemicals (Houston, USA). AU-565 (# CRL-2351), MCF-7 (# HTB-22), and BT-474 (# HTB-20) human breast carcinoma cell lines and ATCC Hybri-care medium (# 46-X) were obtained from ATCC (Manassas, USA). RPMI-1640 high glucose medium (# 30-2001), and RPMI-1640 medium (Gibco # 11-875-135) were purchased from Thermo Fisher Scientific (Waltham, MA, USA). Similarly, HER2 primary mAb (# MA5-13675), goat anti-rabbit HRP secondary antibody (# 31460), goat anti-mouse DyLight 633 secondary antibody (# 35512), and goat anti-rabbit DyLight 633 secondary antibody (# 35562) were obtained from Thermo Fisher Scientific (Waltham, MA, USA); HER3 mAb (# 12708S), phospho-HER2 mAb (# 2247), phospho-HER3 mAb (# 4791), phospho-ERK mAb (# 4370) pan-ERK mAb (# 4695), phospho-AKT mAb (# 9271), pan-AKT mAb (# 4691), cleaved PARP (# 5625), and α-tubulin mAb (# 2125) from Cell Signaling Technology (Danvers, USA), and anti-mouse HRP secondary antibody (# ab6728) were from Abcam (Cambridge, UK). DAPI (#D9542) was procured from Sigma-Aldrich (St. Louis, USA). The pcDNA3.1(+)-TRIM21 mammalian expression vector was procured from OriGene (Rockville, USA) and PULSin^®^ reagent (#501-04) from Polyplus (Illkirch-Graffenstaden, France). Duolink^®^ In Situ Red Starter Kit Mouse/Rabbit (#DUO92101) and Heregulin-β1 (#SRP3055) were from MERCK (Darmstadt, Germany). Human MAPK (# SARB0055) and AKT phosphorylation array (# SARB0017) were procured from Assay Genie (Dublin, Ireland). Low melting agarose (# RM861) was from HiMedia Laboratories (Mumbai, India). Annexin V apoptosis detection kit was procured from BD Pharmingen^™^ (Franklin Lakes, USA), and D-Luciferin (#L-8220) was procured from Biosynth (Staad, Switzerland).

#### TCGA data analysis

cBioPortal for Cancer Genomics (https://www.cbioportal.org/) was used to analyze the TCGA breast cancer patient cohorts’ cancer genomics data. Cohorts were categorized based on DDMs. Their overall survival months and survival status were plotted as the Kaplan–Meier curve. To assess the significance of differences in overall survival months between HER2 mutation- and HER2 WT harboring patients, Mantel-Cox test was used. To evaluate protein activation in MAPK or PI3K-AKT pathways, the TCGA Firehose Legacy dataset and METABRIC dataset were used. Z-score values from RPPA expression data were examined for patients harboring H2-WT, S305C, G309A, S310Y, S310F, and P523S mutation to determine the differences in phospho-protein expression.

#### 3D structure modeling

Human HER2 sequences were retrieved from the Ensembl genome browser and used to develop the extracellular domain model using I-TASSER.^41^ The structures were developed based on the ligand-bound HER2 crystal structure (PDB ID-1S78). The best structures were then mutated using “mutagenesis” tool in Pymol (Schrödinger, Inc., USA). Wild-type and mutant structures were subjected to MDS for up to 1 μs to generate energy-minimized structures and visualize conformational changes resulting from HER2 DDMs.

#### Secondary structure changes owing to dimerization domain mutations (DDMMs)

Amino acid chains of HER2 mutants (S305C, G309A, S310Y, S310F, and P523S) were assessed for potential deleterious effects owing to secondary structure changes using Mupro,^28^ Panther,^26^ and Provean^27^ web server applications.

#### Molecular dynamic simulation (MDS)

To simulate structures of HER2 wild-type and the five point mutations (S305C, G309A, S310Y, S310F, and P523S), molecular systems were generated using GROMACS tool.^42^ Post MD simulation, the trajectories were processed to remove periodic boundary conditions, and RMSD, number of hydrogen bonds, SASA, and radius of gyration were plotted.

#### Docking

HADDOCK server^29^ was employed to study targeted molecular docking on H2-WT and all DDM homodimers using their energy-minimized structures. Residues facilitating HER2:HER2 homodimers were identified from the literature.^3,32^

#### Protein–protein interaction analysis

After simulations, the homodimer interactions of H2-WT and all DDMs were analyzed using the PDBePISA (Proteins, Interfaces, Structures and Assemblies) Web server.^43^

#### Preparation of expression plasmid

The DDMs were introduced using a site-directed mutagenesis approach using the primers included in **Supplementary Table 2.**

#### 3’-UTR knockdown for endogenous HER2

To knockdown endogenous HER2, AU-565 cell line was transduced with high titer pLL3.7-GFP lentiviral particles containing 3’UTR HER2 shRNA cassette (**Supplementary Table 3**) as per standard protocol.^44^ The endogenous HER2 knocked down AU-565 cells were confirmed by western blot and subsequently used further for stable overexpression of H2-WT or S305C, S310Y, S310F, and P523S mutants.

#### Live cell luciferase reporter assay

ZR-75-1 cells expressing H2-WT or DDMs were labeled by the transduction method using Fluc2-tdTomato fusion lentiviral particles. The reporter lentiviral vector was constructed in the lab before using a third generation lentiviral vector.^44^

#### Co-immunoprecipitation

ZR-75-1, MCF-7, and AU-565 were seeded and treated as described in extended material and method section in Supplementary Materials.

#### TRIM-ing method

ZR-75-1 BC cells expressing H2-WT or DDMs cultivated in 35 mm dish as monolayer culture were transfected with TRIM21 expression plasmid using Lipofectamine 2000 reagent. After 24 h of transfection, the HER3 mAb at a concentration of 0.5 μg/μL was delivered using PULSin reagent following an optimized protocol.^37^

#### Proximity Ligation Assay (PLA)

ZR-75-1 and AU-565 cells expressing H2-WT or DDMs were seeded on coverslips and incubated at 37°C. After 24 h, cells were incubated with serum-free media and subsequently treated with heregulin-1β for 15 mins before being fixed with 4% paraformaldehyde. The assay was performed according to the manufacturer’s recommended protocol, using 1:200 dilution for both HER2 (mouse origin) and HER3 (rabbit origin) monoclonal antibodies to check for HER2:HER3 heterodimer. The HER2 (mouse origin) and p-HER2 (rabbit origin) antibodies were used to measure HER2:HER2 homodimer formation.

#### Invasion assay

The invasion assessment was done following a published protocol.^45^ In brief, 25,000 cells/well were seeded in 24-well cell culture inserts (# TCP257, HiMedia, Mumbai) after coating with 250 μg/mL Matrigel (# 354234, Corning, USA). After 16 h incubation, cells were stained with CalceinAM (# C1430. Thermo Fisher Scientific, USA) for microscopic visualization and quantification. Corrected total cell fluorescence (CTCF) value was calculated using the formula:

For inhibitor treatment, cells were pre-treated with either AKT-IV (5 nM), trametinib (5 nM), or tucatinib (0.5× of their respective IC_50_) for 48 h before seeding for invasion assessment.

#### Human MAPK and AKT phosphorylation array analyses

To validate the complete activation of either MAPK or AKT signaling arm in ZR-75-1 cells expressing H2-WT and its DDMs, 500 μg of lysate was used for the array following the manufacturer’s recommended protocol.

#### Orthotropic breast cancer tumor and non-invasive luminescence imaging studies

All *in vivo* preclinical experiments were conducted as per approved protocol (35/2021) by the Institutional Animal Ethics Committee at ACTREC, TMC, India, and were strictly performed in accordance with the accepted guidelines. For implantation, 5×106 re y luciferase reporter-labelled cells (previously mentioned) were injected into the mammary fat pad of 6 weeks old SCID mice. Non-invasive bioluminescence imaging (BLI) scan was performed using IVIS Spectrum (Perkin Elmer) after injecting 100 μL of 30 mg/mL D-luciferin substrate. Mice were maintained under 2% isoflurane gas anesthesia during the scan. Data analysis was performed using Living Image software v4.4 (Perkin Elmer, USA). Tumor volume was measured using a Vernier caliper every 5 days and calculated according to the formula: ½ length × width^2^. Trastuzumab was administered intraperitoneally at 30 mg/kg once every week for 3 weeks. The study was completed at 60 days post-implantation or when the tumor burden reached ~800 mm3, whichever was earlier. At the endpoint, mice were euthanized by cervical dislocation, and ex vivo imaging was done to cross check the occurrence of metastasis. Tumor tissues were collected and processed for lysate preparation according to protocol published in the literature^3^.

### Quantication and Statistical Analysis

#### Statistical analysis

All data are presented as mean ± standard deviation (SD). Statistical significance was assessed using Student’s t-test using GraphPad Prism 8 software (GraphPad Software, La Jolla, CA). P values <0.05 were considered statistically significant, and the CI was set at 95%. Statistical analysis was done for a minimum of three independent biological repeats.

## Data Availability

This paper does not report original code. Any additional information required to reanalyze the data reported in this paper is available from the lead contact upon request.
